# Chronic pain patients’ need for recognition and their current struggle

**DOI:** 10.1007/s11019-021-10040-5

**Published:** 2021-07-14

**Authors:** D. Koesling, C. Bozzaro

**Affiliations:** Department of Medical Ethics, CAU, Kiel, Germany

**Keywords:** Chronic pain, Social aspects of chronic pain, Struggle for recognition, Patients’ experience, Social philosophy

## Abstract

Chronic pain patients often miss receiving acknowledgement for the multidimensional struggles they face with their specific conditions. People suffering from chronic pain experience a type of *invisibility* that is also borne by other chronically ill people and their respective medical conditions. However, chronic pain patients face both passive and active exclusion from social participation in activities like family interactions or workplace inclusion. Although such aspects are discussed in the debates lead by the bio-psycho-social model of pain, there seems to be a lack of a distinct interest in assessing more specifically the social aspects regarding chronic pain. As a result, the social aspects have yet to be taken into a more thorough theoretical consideration of chronic pain and to be practically implemented to help affected patients. By addressing chronic pain patients’ struggle for recognition, this paper attempts to shed light on some of these social aspects. We base this attempt on a theoretical framework that combines patients’ statements with an adaptation of Axel Honneth’s social-philosophical work on *recognition*. Thus, this paper tries to make a suggestion on how the bio-psycho-social model of pain can live up to its name by helping to address more adequately some of the more neglected aspects in chronic pain patients’ suffering than has been possible to date.

## Introduction

Chronic pain patients’ reports about their conditions and experiences reveal that forms of misunderstanding, rejection, and stigmatization greatly shape their suffering. Patients face these experiences of ostracism and isolation in various social constellations. These occur at three levels: privately, within patients’ own immediate surroundings of family, friends or colleagues; institutionally, through interactions within doctors’ or therapists’ offices, public administrations, and/or insurance agencies; and at the societal level where, for example, a patient’s condition puts constraints on their ability to meet current performance standards and expectations. These aspects contribute to the problem of patients’ social exclusion which is an essential part of chronic pain patients’ suffering. However, these aspects are yet to be adequately addressed in research. To do that and grasp the complex social entanglements in the process genuinely social-philosophical approaches appear to be promising as they are able to grasp the complex social entanglements involved.

In this paper we will show that Axel Honneth's work^1^ might be a good fit for this intent. Honneth’s implemented terminological pair of *disrespect* (or *disregard, misrecognition*) and *recognition* not only enables more adequate access to these negative social experiences, but also provides the basis to set up normative obligations on the various social levels, which are “grounded in concrete social experiences rather than abstractly formulated moral principles or principles that are derived a priori and then applied to the world.” (Wilhelm 2021: 53) In the following we first, want to highlight some of the social problems chronic patients face due to their respective conditions. Following McGee et al. (2011: 1383, italics added by the authors) in this regard, who have rightfully stated that “chronic pain ethics needs to be focused upon and framed by the *experiences of people living with chronic pain*.” Building on this, we then adapt Honneth’s theoretical approach and show that chronic pain patients’ situation can be examined within the framework of a *struggle for recognition* by acknowledging social problems in the spheres of primary relations, legal relations, and solidarity. Finally, we make some suggestions on how the framework of a struggle for recognition might help to understand the suffering of patients living with chronic pain and what normative obligates might follow.^2^

## Chronic patients’ experience of social struggles

Comprehensive studies, such as the one by Breivik et al. from 2006, have documented various negative effects chronic pain has on the affected people. These range from them experiencing a change of their own corporeality and the disabling effects of the pain to the disintegration of their previous life plans and lifestyles. When it comes to social aspects, chronic pain patients experience a lowered ability to keep up with friends, maintain a job or partake in social events. These examples stand out as some of the negative impacts chronic pain has on people’s social lives. Additionally, studies have shown that depending on the specific form of chronic pain and the social status, there are variances in not only the very impact the chronic pain has on a person’s life, but also to their possibilities of alleviating their pain (see Mills et al. 2019: e274–e275; Karos et al. 2018; Goldberg 2014: 16–18, 27, 127; McGee et al. 2011: 1383). These findings are in line with the bio-psycho-social model of pain that indisputably exemplifies an existing research interest in social aspects of chronic pain. At present, there is increased research emphasis on those social aspects which are objectifiable, measurable or quantifiable. This is especially so in areas like the interdependence of pain and socio-economic status or the lengthy journeys patients undergo to receive proper health care from specialists. However, the quality of relationships or other, more subjective social aspects, remain rather neglected—even though they have been acknowledged in the predominant perspective in the discourse.

To highlight some of the less regarded aspects, we present the following insights into chronic pain patients’ situations. This serves the purpose of illustrating some of the possible challenges and hardships people enduring chronic pain might have to face due to their condition. We try to combine our analysis with patients’ own view and reflections on their situation as we share Jackson’s (2000: 15) assessment, that “[w]hat they have to say is worth hearing and it should be given greater weight in the effort to understand the lived reality of severe chronic pain.” Certainly, there is no doubt that the considerations we present cover only some neglected aspects and of course, are not applicable for every single individual who suffers from chronic pain. However, they do represent aspects which are shared even amongst some of the people suffering from other chronic diseases but that are common and widely shared amongst chronic pain sufferers. As e.g. Scarry (1987: 4) has emphasized, pain is always evident to the one bearing it, but it is not for the one not bearing it. Often only metaphoric imagery, such as feeling *like* being stabbed or *like* having a burn, when there is neither an actual stabbing nor a burn happening, can “be used [to] associatively […] express pain.” (Scarry 2007: 13) There has been a long and on-going debate on how and in which way pain is properly communicable, but in a generalizing way one can say that pain is intersubjectively elusive, because “[t]o have pain […] means to have unmistakable certainty and yet to be essentially alone with the concrete form of this undoubted experience.”^3^ (Kohlmann 1997: 136; see 136–138; Edwards et al. 2014: 367). This creates a huge problem for those not suffering chronic pain, as usual pain indicators such as wounds, are misleading or missing here. One of the main shared social aspects in patients’ experience with chronic pain is connected to the various conditions’ generally being invisible. As e.g. Jackson (2005: 340; see 2000: 37–38) stated rightfully, “the lack of a visible mark is considered by some pain sufferers to create the conditions for stigmatization. Severe chronic pain is delegitimized in several ways, all serving to question the pain's reality.” This interlinks to an impossibility of making many forms of chronic pain visible via imaging procedures like MRI or CT scans. This fact renders chronic pain a *contested* or *controversial disease*, as the patients’ experiences and claims cannot be made easily comprehensible for others.^4^ One patient describes the situation the following way:

I think it's hard for anybody. The basic problem is that I look perfectly normal, you know, to all intents and purposes I can do most things. I can drive a car, I can do the shopping, I can meet trains. I mean I can, I look perfectly normal. I walk, I could walk upstairs. Okay I pull on the handrails to get upstairs but to... I'm a complete physical specimen. If I'd lost a leg, one often feels that pain, people with chronic pain often use a walking stick because they want people to understand that there's something wrong and that it can't be seen. Pain can't be seen (healthtalk.org w. y.; see Scholz 2010).

As a result, patients are often suspected of exaggerating or even lying about their condition, sometimes even leading up to have suicidal thoughts, plan, attempt or even commit suicide (see Jackson 2000: 38, 44–45). Consequently, lay people and also health care professionals frequently raise doubts about the chronic pain’s severity or even its existence. Therefore, as a medical condition, chronic pain is “typically at greater risk of skepticism and suspicion” (Goldberg 2014: 52, see 75, 124, 127; see Gjesdal et al. 2018: 518; Williams 2016; Grüny 2004: 169–170). This creates an atmosphere of disbelief and lack of trust in a patient’s relationships with their family, peers or health care professionals. Chronic pain patients thus often feel helpless, invisible, ignored, and undermined in their experiences of pain. This is because they are being branded as exaggerators or even malingerers and confronted with stress, harmful pejorative stereotypes, and humiliating stigmata. These include being labelled as freeloaders of the medical system or the social security system (see Karos et al. 2018: 1690; Gjesdal et al. 2018: 521–522; Edwards et al. 2014: 373; McGee et al. 2011: 1383; Thomas 2000: 684). Such drastic doubt about chronic pain patients’ honesty and authenticity regarding their condition becomes quite problematic for the patients, because, “social support, rest and affection [which] can reduce the pain or make it bearable, by taking the patient as a person in need and not as a committed participant or leave them to themselves in their helplessness”^5^ (Grüny 2004: 159, authors’ translation; see MacDonald and Leary 2005: 217). Research also indicates that “higher levels of social support are associated with lower levels of chronic pain” (MacDonald and Leary 2005: 207).

In line with these arising doubts about chronic pain patients, chronic pain itself is often interpreted as a personal failure or flaw rather than a serious medical condition that needs treatment, help or assistance. While acute pain usually evokes forms of fond attention and the affected people perceive an attitude of care by those surrounding them, this is frequently not the case for chronic pain patients. Here, skepticism is predominant and the chronic pain patients tend to face “a culture of stigma and distrust, and find their dignity undermined by a system and a society that appears disinterested in taking a stand for the care and consideration to which they are entitled.” (McGee et al. 2011: 1383) These outlined social struggles of chronic pain patients become tangible in Kleinman’s (1988: 57) summarizing statement: “If there is a single experience shared by virtually all chronic pain patients it is that at some point those around them—chiefly practitioners, but also at times family members—come to question the authenticity of the patient’s experience of pain.”

Although Kleinman’s statement dates back over 30 years it remains true today in the eyes of many affected people. Thus, while some patients express great satisfaction regarding their (medical) treatment and care, others feel like they are not being taken seriously with their issues and needs, as Gjesdal et al. (2018) recently illustrated. Evidently these insights into chronic pain patients’ experiences show a common lack of knowledge about the respective conditions and chronic pain’s limiting and restricting effects on the affected people (see MacDonald and Leary 2005: 216). Additionally, those insights mark a gap between pretensions or aspirations held by people in patients’ surroundings and health care professionals and the patients’ actual capabilities and possibilities that are diminished by the chronic pain condition itself. This can lead to what patient A. Wagner (2008, authors’ translation, italics added by the authors; see Jackson 2000: 45) describes as “the experience that the healthy and the ill live in *different worlds*. Simply because the rules and the standards just aren’t the same.”^6^

Such a separation between chronic pain patients and the people they interact with poses a problem for the patients. People suffering chronic pain depend on support that only other people can provide, yet others frequently deny that assistance, and thus, create “a lack of recognition” (Gjesdal et al. 2018: 521). Simply viewing this as just a form of disrespect would underestimate the related, extensive consequences, which Taylor (1994: 25; see 26) pinpoints in his research: “Nonrecognition or misrecognition can inflict harm, can be a form of oppression, imprisoning someone in a false, distorted, and reduced mode of being.” For this reason, a conflict of recognition arises in the context of chronic pain, as “quite apart from the existential meaning of an understanding of what is involved in the intolerable processes of one's own body, the social *recognition* of the suffering is essential” (Grüny 2004: 146–147, authors’ translation, italics i.o.).^7^ In case this recognition is lacking, the chronic pain patient’s suffering can neither be understood nor alleviated. If understanding and alleviating the suffering of chronic pain patients is an aspired goal for both medicine, where it is a constitutive factor, and society in general, a more elaborate understanding of this conflict of recognition itself is required.

Being aware of the social recognition mentioned above is a rather complex phenomenon that ranges from the micro- to the macro-level of society. Therefore, some controversies or disputes regarding the acknowledgement of chronic pain are occurring. These can be made easily visible by e.g. accounting for the activities of the various support groups that are especially concerned with chronic pain. Support groups are not only a vital institution of mutual help through networking, sharing information and knowledge, and having meetups and conferences. They are also a joint voice of those affected by chronic pain in the political discourse. Thus, for example, the support group *Deutsche Schmerzliga e. V.* (n. d., authors’ translation) “advocates the right of patients to have competent treatment. (…) [and] campaigns to increase policymakers and the general publics’ understanding about the problems people with chronic pain face.”^8^

This indicates that the conflicts surrounding the recognition of chronic pain do not only take place in dyadic doctor-patient-relationships, or in smaller circles of family, friends, and colleagues. Rather, these conflicts also extend to organizations and institutions and to the stage of health policy and economics. This becomes evident as well when tracing patients’ journeys and the importance and need of a (specific) medical diagnosis by their doctors. Such a diagnosis does not only *authenticate* a patient’s condition in front of their peers, but also the health care systems or pension funds, which influences their financial status strongly.^9^

## The struggle for recognition of chronic pain patients

Considering the preceding outlines, it should already be clear that the depicted phenomena can be described as a struggle for recognition in a more general way that is in line with a trend of the last several years to frame many social conflicts as conflicts of recognition (see Fraser and Honneth 2015: 7; Fraser 2015: 15–27). However, the depicted phenomena can furthermore be transferred or translated in a more specific way as a *struggle for recognition* that takes up a theoretical framework provided by more recent discussions in social philosophy. As it is neither possible nor necessary here for the argument’s sake to reconstruct those complex academic debates about recognition, a reference to Honneth’s work should suffice as a starting point for further presenting the idea we pursue here.^10^ His work seems most applicable as a starting point, as it not only has an explicit and genuine societal-theoretical point of view, but has already been successfully applied and discussed in various contexts, such as Salminen’s (2020) recent study regarding young people and professionals in social and youth work. But as Honneth has not specifically addressed the topic chronic pain, we will need to make some necessary conceptual adaptations.

In *The Struggle for Recognition* (Honneth 2005), Honneth combines ideas from Georg Wilhelm Friedrich Hegel’s early work on this topic with the work of George Herbert Mead to reestablish the term *recognition*. Honneth makes use of this term to describe a fundamental aspect of social life, highlighting the social interdependency of one human being to a counterpart as a constitutive condition for subjectivity and as an enabling condition for a successful life or even a good one. In this manner, he presented recognition quite literally as “a vital human need” (Taylor 1994: 26), because “[i]n order to acquire a successful relation-to-self, one is dependent on the intersubjective recognition of one’s abilities and accomplishments.” (Honneth 2005: 136) Hence recognition is not only conceptualized as something positive, but even further, something necessary for the individual. When understood this way, recognition is not merely cognizing another human being, but rather “the expressive act through which this cognition is conferred with the *positive meaning of an affirmation*” (Honneth 2001: 115, italics added by the authors).

However, this recognition does not simply exist and “[a]ll types of practical self-relations are fragile in the sense that, once formed, they continue to be vulnerable to the erosive effects of misrecognition.” (Wilhelm 2021: 65) Recognition hence must not only be established, but also maintained via reciprocal, conflictual action, as Honneth’s usage of the term *struggle* indicates. At the heart of such struggles is the absence, denial or lack of desired recognition. This is labeled as *disrespect,* which, contrary to everyday language “refers not merely to a failure to show proper deference, but rather to a broad class of cases, including humiliation, degradation, insult, disenfranchisement, and even physical abuse” (Anderson 2005a: iix; see 2005b: xviii). In Honneth’s understanding, this renders the term disrespect quite specifically as “an injustice not simply because it harms subjects or restricts their freedom to act, but because it injures them with regard to the positive understanding of themselves that they have acquired intersubjectively.” (Honneth 2005: 131) In this perspective there is a normative obligation to try to minimize disregard, as it is a condition that people intensely dislike and one that ought not to exist. At the same time, attempts must be made to establish all forms of recognition. In this sense recognition and disrespect are complementary, whereas a denial of recognition leads to disrespect which obstructs the possibility of a good life as it disrupts a person’s process of identity-formation. Overall disrespect “hinders or destroys persons’ successful relationship to their selves.” (Iser 2019).

Applying one overarching perspective that follows yet transforms Hegelian and Meadian thought demonstrates that Honneth (2005: 131–139; see Bedorf 2010: 47–63) is concerned with various forms of normatively substantial disrespect appearing in three different types of social spheres that he distinguishes in bourgeois society. These are: (1) the sphere of primary relationships or sphere of love and friendships, where disrespect prominently takes on forms of physical abuse; (2) the sphere of legal relations, where disrespect appears in forms such as exclusion or denial of rights; and (3) the sphere of solidarity where disrespect takes on forms of denigration or humiliation. For now, this proves to be a rather abstract differentiation that seems to be more of an analytical distinction than a radical one. We highlight subsequently how this can be connected with the specific topic of chronic pain and the affected persons’ social hardships.

### The sphere of primary relationships and the withdrawal of attention

In the sphere of primary relationships, one can identify the most fundamental mode of recognition. What Honneth describes here as love refers to a way of caring for one’s basic needs by significant others. In the insinuated sense, *love* is not restricted to romantic love by spouses, significant others, etc., but is to be understood in a broader sense including all meaningful connections on an emotional level. In this sense one should mistakenly narrow *love* as it is terminologically coined here to “describe[] an emotional relation to others that achieves social integration of concrete individuals through acknowledgement of and care for emotional and physical needs and desires.” (Wilhelm 2021: 56) Hence, “[l]ove relationships are to be understood here as referring to primary relationships insofar as they—on the model of friendships, parent–child relationships, as well as erotic relationships between lovers—are constituted by strong emotional attachments among a small number of people.” (Honneth 2005: 95; see Deines 2007: 279) Employing an ontogenetical line of argument (based on the parent–child relationships), these kinds of relationships are fundamental for human beings as they are constitutive for any self-confidence an individual can develop. Additionally, although there are socio-cultural variations, *love* is an *ahistorical* precondition for self-confidence (see Honneth 2005: 107, 133). In this sense, the kind of recognition provided in the sphere of primary relations is prior to all other forms and spheres of recognition. Prime examples of disrespect in the sphere of primary relations for Honneth are torture and rape, which combine physical pain, loss of power, being overpowered, abuse, and helplessness. In such cases of “mistreatment, torture and rape the perpetrators do not only intentionally inflict pain and injury on their victims but also deride the agency of the latter.” (Iser 2019) Exploiting and condoning pain or an intention of actively inflicting it is constitutive for this specific kind of disrespect, where the physical integrity of the sufferer is endangered by others and physical as well as psychological well-being might get destroyed (see Deines 2007: 280).^11^

Adapting this for chronic pain creates the need for a particular theoretical alteration,^12^ as the disrespect in this sphere does not inherently connect to the chronic pain. Rather, it is more often an *incidental byproduct* of the pain condition itself. Here chronic pain patients’ physical integrity has been damaged already. This damage, though, is not necessarily^13^ a result of a voluntary, abusive act by another person, but due to medical conditions. This makes it different in its genesis than Honneth’s examples. However, it is not uncommon that, family members, friends, coworkers, and peers start to turn away from the chronic pain patients or abandon them completely in the context of the chronic pain patients’ condition.^14^ Thus, the patients’ need for caring companionship is neglected. There are evidently forms of disrespect towards chronic pain patients which fit a Honnethean framework in the primary relationships, although these may be more subtle than in other cases. Situations such as being desperate for a gentle hug, not being lent an ear or being socially absent from their closest surroundings evokes forms of suffering, especially forms of social isolation. Patient P. Andresen (2009, authors’ translation) described it quite simply:

Our environment today is geared to functioning and hence, there is simply no demand for people with pain. And they are no longer nice to have around and then they are alone quickly. [...] Then comes the unfortunate realization: If you are lucky and one person stays there, or two, that is quite a lot then. And you cannot expect that either.^15^

Such a withdrawal of dependable and reliable companionship does not only add another dimension to the pain experience itself. The retraction of significant others, regardless of whether it is a slow process or an abrupt loss, can result in a hurtful isolation for the affected people. They have not been accepted in their condition by the people who are closest to them. Although it is understandable and desirable—even from a non-subjective, normative standpoint—if chronic pain patients could be part of loving relationships, one has to acknowledge that love is something that cannot be imposed or enforced. As Wilhelm (2021: 57) recently put it: “Love as deep affection cannot be expanded at will or to too many individuals” and therefore one cannot simply be asked to love someone.

### The sphere of legal relations and conflicts about the enforcing of rights

The second sphere, the one of legal relations, presents itself as distinguished from the sphere of primary relations. This sphere is historically *contingent,* and in and of itself, a product of history. One attains recognition here explicitly not as a specific individual, but as an equal member of the general public. By institutionalizing rules via legal practices in legislative procedure or organizations like independent courts, the legal relations guarantee a person’s freedom and status as an equal without considering their specific individuality. By being granted legal *rights* and being able to act according to them, a person is not only able to establish self-respect, but also learns to reciprocally take the point of view of the other and respect the other (see Honneth 2005: 133, 1990: 1049; Deines 2007: 280). Having legal rights is contingent, so controversies arise about the central question, who obtains them and is therefore a person in a *moral sense*. Depending on how this question is answered and who is included and excluded in the understanding of a person in a moral sense leads to quite a difference in which rights are guaranteed to whom and to what extent. In line with this, the withholding or withdrawal of recognition here “refers to those forms of personal disrespect to which an individual is subjected by being structurally excluded from the possession of certain rights within a society.” (Honneth 2005: 133) This consequently leads to “the feeling of not enjoying the status of a full-fledged partner to interaction, equally endowed with moral rights” (Honneth 2005: 133; see Deines 2007: 281). This damages a person’s self-respect.

One example of such disrespect in the legal relations is the exclusion of people because of their heritage, sexuality, religion, or skin color. In such cases, a person is denied the legal entitlement due to contingent empirical factors and there is a misapplication of rights or a refusal to apply them at all (see Wilhelm 2021: 82). In a similar way, some chronic pain patients seem to feel that they are denied legitimate claims. This conflict, next to the documented “disparities in access to pain care for certain groups” (Edwards et al. 2014: 373; McGee et al. 2011: 1377–1378, 1381–1382) and whether due to financial, geographical or other reasons, can be identified in a disparity in the interpretation of what chronic pain patients are rightfully owed and what they need for an actual improvement of their situation e.g. in forms of payment for medical treatment in rehabilitation programs. In this context, patient I. Meyer (2009, authors’ translation) stated: “And according to the law, it all sounds very different. When I read the statute book now and then I think: well my God, you have actually taken care of it, you will find help everywhere, but in practice it is not so.”^16^ Such a difference between patients’ perception of what they deserve and the institutionalized way of aiding them, as well as the interrelated conflicts can become virulent. Patient I. Meyer demonstrated this when explaining about the complex processes of evaluation and revaluation when it comes to official assignment of care levels: “You have to fight for everything. I had care level two, now I am to be downgraded to one, even though I felt worse. Yes, so then I was informed, care level one, […] then I had to go to battle again, now it is in the works, and afterwards it should then be decided” (Meyer 2009, authors’ translation).^17^ Such an exhausting and bureaucratic task is only one aspect of the conflict, though, as it is often accompanied by stigmatization due to the partial or whole denial of their legal claims.

### The sphere of solidarity and the limited capability to contribute to society

The third sphere Honneth addresses is the one of solidarity, which like the sphere of legal relations, is also a historical product. However, there is a central difference. In contrast to the legal relations sphere, in the sphere of solidarity, one does not receive recognition as a result of being a person in a moral sense and hence, on the basis of a generalized concept. Instead, recognition here is based on a shared set of specific values the members of the sphere of solidarity agree on. When a person matches and satisfies those specific values and therefore contributes to common goals, they are recognized as a valuable member in the shared social praxis and can establish self-esteem. As one’s self-esteem relies heavily on contributing to and participating in society in a culturally sanctioned way, a problem for the individuals can arise “[i]f this hierarchy of values is so constituted as to downgrade individual forms of life and manners of belief as inferior or deficient, [because] then it robs the subjects in question of every opportunity to attribute social value to their own abilities” (Honneth 2005: 134; see Deines 2007: 281). This value of one’s specific capabilities can vary as the values themselves are contingent. They are “the result of social and cultural struggles that lack the universality that is distinctive of legal relations” (Anderson 2005b: xvii).

Modern society contains many values, such as legal equality or helping people in need. However, nowadays performance—especially in the work context—seems to have become a main priority, or core value. This has an excluding impact on various groups of society. The elderly comprise one such group because as they age, elderly people can have the feeling that they cannot contribute anything valuable to society when they are no longer part of the economic production system. Many chronic pain patients have the same feeling of being unable to contribute to society, as they cannot fit within the dominant norms of performance, productivity, and efficiency. Their pain condition hinders them from working fully or even working at all and “[s]ome who continued to work found they could no longer perform adequately. Others said they worked in constant pain.” (Jackson 2000: 35) As a result of such limitations, chronic pain patients are often no longer perceived as fellow human beings who, despite their limitations, may be able to contribute something valuable to society. Instead, they are often exclusively viewed as supplicants or claimants, although they actually want to partake, but are simply unable due to their condition.

Patient Klein’s (2009, authors’ translation) statement is a testimony of this kind of social struggle: “And I don't have to throw myself into the job market because nobody would hire me anymore. No, I can kiss that goodbye […] [Y]ou're not that old at all and suddenly the world has no use for you. And then I really don't know what to do. I really do not know. I lack my place in this world.”^18^ Not only do such statements express feelings of despair and uselessness, they also reflect that some of those affected by chronic pain cannot perceive themselves as a valuable contributor to the community and are not given the desired social appreciation. They are structurally excluded by not matching societies’ norms of contributing—especially economically. Often, they are only acknowledged as chronic pain bearers and not as appreciative human beings who are able to partake in society despite their pain-induced restrictions. In this way, chronic pain patients are excluded from societal participation.

## Summary

This article aimed to present in more detail the social aspects of chronic pain that are acknowledged in the bio-psycho-social model of pain, but have not yet been profoundly elaborated. Hence, the understanding of the social circumstances of chronic pain, its meaning for the patients’ lives, and the social consequences that are often invisible and have a subtle impact, lags behind other research areas. We began by presenting some established research that addresses social aspects of pain and the widely accepted knowledge that social exclusion itself can be hurtful. Then, we provided some insight into chronic pain patients’ experience to illustrate some of the social problems that arise, and subsequently lead to a conflictual social embeddedness resulting in a struggle for recognition. By building on some of Honneth’s reflections on disrespect, it became possible to translate various aspects of these conflicts into social philosophical terminology.

A perspective on chronic pain like we have attempted here, might help by highlighting an absence of social support on various social levels, resulting in various forms of isolation, exclusion and dismissal (see Kieselbach et al. 2016: 352–353; Newton et al. 2013: 166–167; Jackson 2000: 38). It illustrates the different areas of the social conflicts and how they might need addressing to alleviate the suffering of chronic pain in its social regard, which Buchman et al. (2017: 36) hint to as well when they state: “A desire to avoid ‘pervasive distrust’ may be what really matters to persons living with pain, as they strive for their claims to be considered credible and their character considered trustworthy.” Describing the situation of chronic pain patients with the help of the terminology and ideas involved in texts such as *The struggle of recognition* helps to make chronic pain visible in new ways. This also gives voice to “perceived relational devaluation resulting from a pain disorder [which] might add directly to an individual’s suffering” (MacDonald and Leary 2005: 217). The approach we presented might help to consider how to properly address and also narrate the topic of chronic pain in various academic debates so that all participants recall,

that for many pain sufferers, their lived experiences are marked by isolation, stigma, and exile, even from their own bodies. Thus […] the primary means of alleviating the pain patient’s existential suffering is to bring them back into the social world and into a sense of community. […] Even the very act of listening to the pain sufferer and taking a narrative can be an important step in bringing the pain sufferer into society (Goldberg 2014: 86).

Building on the various forms of disrespect and the interconnected struggles for recognition might therefore be very useful in understanding chronic pain better as a whole, since an experience of disregard such as “[t]he psychological pain of being disbelieved and stigmatized is surely as devastating to these patients as their bodily pain, perhaps more so” (Thomas 2000: 697). Thus, such an attempt is not only a theoretical gimmick, as “the experience of being disrespected carries with it the danger of an injury that can bring the identity of the person as a whole to the point of collapse” (Honneth 2005: 131–132). Neglecting this in the context of chronic pain will lead to a failure to properly conceive the complex suffering chronic pain can create. As McGee et al. (2011: 1383; see Cassell 2012) noticed with inadequately treated chronic pain patients, they suffered “not only physically but in all aspects of personhood.” Hence, helping chronic pain patients does not only mean alleviating the pain experience itself. It can consist—and when alleviation is not possible, must consist—of easing the suffering connected to chronic pain by means of simply spending time with the affected person. Listening to and talking with them acknowledges that the chronic pain patient is still being a human who is deserving and worthy of respect, affection, and care (see Frede 2018: 14). This results in recognizing that chronic pain is not simply another problem that just needs medical-pharmaceutical attention, but also explicit treatment as a social problem, which, as we have shown here, connects with “experiences of exclusion, alienation, rejection, and powerlessness” (Edwards et al. 2014: 371).

As indicated above, one central consequence of this is that chronic pain cannot be understood as a problem that falls exclusively into the area of medicine, or the field of psychology or the psychosomatic: “As important as medical services are to ameliorating disease and human suffering, medical care is neither intended to nor generally equipped to address the macrosocial factors that determine patterns of pain and its distribution in populations.” (Goldberg and McGee 2011) Health care professionals are, of course, absolutely necessary to provide some (forms of) relief from chronic pain and find ways to deal with the chronic pain and its extensive consequences. Social aspects of chronic pain can only be adequately addressed by people who recognize the chronic pain patients in their daily struggles and help them to bear the burdens, yet do not fall into the pattern of viewing the patients solely as sick people. However, these professionals cannot attend to all aspects by themselves, which becomes comprehensible when comparing forms of recognition which can be expected in a professional setting compared to those which cannot be expected in the same way, such as love. Hence, one must restrain from to delegate the complex normative challenge to medical personal and misinterpret it as their sole normative obligation or duty to help chronic pain patients. Not all forms of recognition can be provided by single groups of persons. Family and friends provide different forms of support than medical personal and vice versa, and they are not simply interchangeable.

These is a general need for people, whatever profession or specific role they have in chronic pain patients’ lives may be, to help those with chronic pain maintain an active role in society and retain control of the social aspects of their lives as much as possible. As Karos et al. (2018: 1690) noted, “pain [undoubtedly] places the individual in a state where they rely on others for support and transferring control to others such as family, friends, and most importantly, health care providers.” This perspective of recognition presents a serious normative obligation: Try to treat chronic pain patients with as much care and equality as possible while considering patients’ individual restrictions and being supportive, not patronizing. This should not be mistaken to simply accept chronic pain patients’ perspective without challenging it in any way. It rather has to be a genuine and unbiased effort to understand and help the patient in his complex live-changing affect chronic pain often is, but this does neither imply to just simply do everything a patient asks nor a simple affirmation or echoing of patients’ view of their pain, but to support him or her. That can also mean to engage with chronic pain patients’ condition, situation, feelings and needs in a critical, yet benevolent way. This is important as it exemplifies that, “[n]either every negative description of a person(’s self-image) nor every challenge of her current status position—as hurtful as such ‘challenges’ might be for the affected person—is necessarily a form of misrecognition. Quite to the contrary, only by being subject to well-meaning criticism can we improve ourselves.“ (Iser 2019).

Finally, we recognize that on the institutional level, more efforts are needed to support chronic pain patients manage a life with chronic pain better. Implementing health literacy programs and other forms of inclusive projects could help achieve this goal and provide chronic pain patients with the recognition, support, and visibility they so greatly need. The paper primarily approached the problem with a perspective on the chronic pain patients. Yet this should not be mistaken to exclude the macrosocial context aspect the subjects are embedded in. There can be no doubt, that this calls for a macrosocial perspective and we were only able to provide some insight into this here. Hence, a “shift in policy from the micro-level focus on the provision of health care services to a macro-level approach addressing the structural determinants of health dovetails with a number of practices and reports urging the same kind of health policy shift” (Goldberg and McGee 2011) is essential to understand the complex situation of chronic pain patients better. These aspects should be investigated further with a more complex analyses of social norms in modern, mostly capitalist societies, their mechanisms and structures, as “[t]he daily agony of individuals with chronic pain is often intensified by the way this larger society views their predicament and attempts to deal with it.” (Jackson 2000: 2) This calls especially for further research dedicated to the link between power, norms and discrimination which is as play in the case of chronic pain.

## Footnotes

1 This paper mainly refers to Honneth’s *The Struggle for Recognition: The Moral Grammar of Social Conflicts* and is not primarily concerned with the identifiable and challenging modifications or changes in his work throughout his academic career, which e.g. Wilhelm (2021: 57) or Haber (2007) have commented on.

2 The statements are taken from healthtalk.org and krankheitsheiterfahrungen.de, which consist of interviews with sufferers of various illnesses or diseases like chronic pain. The authors have translated statements originally given in German into English and done so as literally as possible, while trying to keep the specific tone and notion of the statements. For the quotes taken from academic literatures used, e.g. Honneth’s work, we referred to official English translations where they are available. Where such translations are not available the authors have translated relevant passages as described to provide transparent and hopefully adequate translations. In all cases, we provide the original quotes in German as footnotes.

3 “Schmerzen zu haben […] heißt untrügliche Gewißheit zu haben und doch mit der konkreten Gestalt dieser unbezweifelbaren Erfahrung wesentlich allein zu sein.” This very idea is in line with a recent phenomenological definition of pain provided by Geniusas (2020: 8, italics i.o.): “*pain is an aversive bodily feeling with a distinct experiential quality, which can be given only in original firsthand experience, either as a feeling-sensation or as an emotion*.”

4 In this respect chronic pain is very similar to some psychiatric diseases like depressive disorders.

5 “daß sozialer Rückhalt, Ruhe und Zuwendung den Schmerz reduzieren oder erträglicher machen können, indem sie den Patienten als Bedürftigen und nicht als verpflichteten Teilnehmer nehmen oder in seiner Hilflosigkeit sich selbst überlassen”.

6 “die Erfahrung, dass Gesunde und Kranke doch in verschiedenen Welten leben. Einfach weil die Regeln und die Maßstäbe einfach andere sind.”

7 “Ganz abgesehen von der existenziellen Bedeutung eines Verständnisses darüber, was es mit den unerträglichen Vorgängen am eigenen Körper auf sich hat, geht es hier wesentlich um die gesellschaftliche *Anerkennung* des Leidens”.

8 “Die Deutsche Schmerzliga hat das Ziel, die Lebensqualität von Menschen mit chronischen Schmerzen zu verbessern. Sie vermittelt Informationen über die Möglichkeiten der modernen Schmerztherapie, über spezialisierte Therapeuten und macht sich für das Recht der Patienten auf eine kompetente Behandlung stark. Sowohl bei den politisch Verantwortlichen als auch in der Öffentlichkeit wirbt die Deutsche Schmerzliga um Verständnis für die Probleme von Menschen mit chronischen Schmerzen.”

9 If chronic pain were recognized as a (independent) disease, this might, as Conrad and Muñoz (2010: 21–22) discussed, possibly be a case of medicalization—which as a phenomenon is often criticized for good reasons—that would benefit those affected.

10 The topic of recognition is already prominent in German Idealism. If one follows contemporary work like Bedorf (2010), for example, there are four types of recognition: intercultural recognition, intersubjectivistic recognition, subjectifying recognition, and misjudging recognition.

11 As should be clear from the explanation the term disrespect is coined in a very specific sense that might differ from everyday language.

12 While an argument can be made that Honneth’s examples of torture and rape focus specifically on intentionally inflicted injuries, he insists that the point he wants to make “is not the purely physical pain but rather the combination of this pain with the feeling of being defenselessly at the mercy of another subject, to the point of feeling that one has been deprived of reality” (Honneth 2005: 132). This seems plausible here because the *effects* of the others’ behavior in the context of chronic pain are *pain*ful, even though they might not be the result of an intentional attempt to hurt one’s (bodily) integrity. This does lead, though, to the loss of the patient’s autonomy of their own body. Additionally, they fall victim to emotional disintegration by their closest ones refusing to offer the patient adequate social support for their medical condition.

13 Yet there are cases where chronic pain can be attributed to others’ doing. A prime example for this is chronic pelvic pain resulting from being abused and raped.

14 Although chronic pain might be one central cause of such an abandonment, one should restrain from a simple monocausal hypotheses here as chronic pain can be one important, but not the sole factor for isolation.

15 “Unsere Umwelt ist heute auf Funktionieren eingestellt und da sind Menschen mit Schmerzen einfach auch nicht gefragt. Und sie sind auch nicht mehr schön um sich zu haben und Sie sind dann schnell allein. […] Denn leider muss man dann feststellen: Wenn man Glück hat und einer bleibt da, oder zwei, dann ist das schon eine ganze Menge. Und man kann das auch nicht erwarten.”

16 “Und laut Gesetz klingt das alles ganz anders. Wenn ich jetzt das Gesetzbuch lese und dann denk ich: naja Gott, da hast du eigentlich ausgesorgt, da findest du überall Hilfe, aber in der Praxis ist es nicht so.”

17 “Man muss um alles da kämpfen. Jetzt ging es wieder um die Pflegestufe. Ich hatte Pflegestufe zwei, jetzt soll ich auf eins zurückgestuft werden, nachdem es mir aber jetzt schlechter geht. Ja, so dann hatte ich Bescheid bekommen, Pflegestufe eins, […] Und ja dann hab ich auch mich da wieder stark gemacht, nun ist es in Arbeit, nun soll diese Sache und danach soll es dann entschieden werden”.

18 “Und auf den Arbeitsmarkt brauche ich mich gar nicht reinschmeißen, weil mich würde kein Mensch mehr einstellen. Nein, das kann ich abhaken. […] [S]o alt bist du gar nicht und auf einmal hat die Welt keine Verwendung mehr für dich. Und ich weiß dann echt nicht, was ich machen soll. Ich weiß es wirklich nicht. Mir fehlt mein Platz in dieser Welt.”
